# Expression and functional analysis of the lysine decarboxylase and copper amine oxidase genes from the endophytic fungus *Colletotrichum gloeosporioides* ES026

**DOI:** 10.1038/s41598-017-02834-6

**Published:** 2017-06-05

**Authors:** Xiangmei Zhang, Zhangqian Wang, Saad Jan, Qian Yang, Mo Wang

**Affiliations:** 1 College of Plant Science and Technology, Huazhong Agricultural University, Wuhan, Hubei 430070 People’s Republic of China; 2 Key Laboratory of Combinatorial Biosynthesis and Drug Discovery (Wuhan University), Ministry of Education, and Wuhan University School of Pharmaceutical Sciences, Wuhan, 430071 People’s Republic of China

## Abstract

Huperzine A (HupA) isolated from *Huperzia serrata* is an important compound used to treat Alzheimer’s disease (AD). Recently, HupA was reported in various endophytic fungi, with *Colletotrichum gloeosporioides* ES026 previously isolated from *H*. *serrata* shown to produce HupA. In this study, we performed next-generation sequencing and *de novo* RNA sequencing of *C*. *gloeosporioides* ES026 to elucidate the molecular functions, biological processes, and biochemical pathways of these unique sequences. Gene ontology and Kyoto Encyclopedia of Genes and Genomes assignments allowed annotation of lysine decarboxylase (LDC) and copper amine oxidase (CAO) for their conversion of L-lysine to 5-aminopentanal during HupA biosynthesis. Additionally, we constructed a stable, high-yielding HupA-expression system resulting from the overexpression of *CgLDC* and *CgCAO* from the HupA-producing endophytic fungus *C*. *gloeosporioides* ES026 in *Escherichia coli*. Quantitative reverse transcription polymerase chain reaction analysis confirmed *CgLDC* and *CgCAO* expression, and quantitative determination of HupA levels was assessed by liquid chromatography high-resolution mass spectrometry, which revealed that elevated expression of CgLDC and CgCAO produced higher yields of HupA than those derived from *C*. *gloeosporioides* ES026. These results revealed CgLDC and CgCAO involvement in HupA biosynthesis and their key role in regulating HupA content in *C*. *gloeosporioides* ES026.

## Introduction

Huperzine A (HupA) is a pyridine-type alkaloid derived from *Huperzia serrata* ^1, 2^ and constitutes a highly active acetylcholinesterase inhibitor, making it a valuable therapeutic option for the treatment of Alzheimer’s disease (AD)^3, 4^. Currently, >46 million people are afflicted with dementia, with this number predicted to increase to 131.5 million by 2050^5^. HupA is highly selective and exhibits low toxicity, reversibility, and a long duration time relative to other drugs used to treat AD^6^. Furthermore, HupA also exhibits anti-inflammatory activity and appears effective in the treatment of cerebrovascular-type dementia and benign senescent forgetfulness^7, 8^.

Currently, HupA is a compound used in herbal supplements mainly extracted from the Chinese club moss *H*. *serrata*; however, it has a limited distribution and slow growth rate^9^. Furthermore, the complex extraction process from plants and the high costs of downstream purification have impeded HupA utility^10, 11^. Consequently, for successful commercial production of HupA, large volumes of *H*. *serrata* are required. Therefore, in order to protect plant resources from over-harvesting and reduce the cost of HupA-containing medicine, alternative methods for mass producing HupA are needed. The chemical synthesis of HupA was attempted, but the resulting synthesized HupA constituted a racemic mixture exhibiting much less potency than natural HupA. Alternatively, some endophytic fungi associated with *H*. *serrata* are capable of producing HupA^12–14^ with *Colletotrichum gloeosporioides* ES026 yielding 45 μg/g dried mycelium according to our previous study^15^. However, HupA production by these endophytes is hindered by low yields and the loss of biosynthetic capability after several generations. Therefore, methods involving overexpression of the enzymes associated with HupA biosynthesis need to be developed in a heterologous host if stable and efficient production is to be achieved^15–17^.

Although HupA biosynthesis remains poorly understood, previous studies revealed its initiation by the decarboxylation of L-lysine to generate cadaverine, with the subsequent formation of 5-aminopentanal. Conversion of L-lysine to cadaverine and cadaverine to 5-aminopentanal is catalyzed by lysine decarboxylase (LDC) and copper amine oxidase (CAO), respectively^18–20^. LDC and CAO were annotated and confirmed as the first enzymes known to participate in HupA biosynthesis in *H*. *serrata* ^17^, and *de novo* RNA sequencing of *C*. *gloeosporioides* ES026 performed by Zhang *et al*.^21^ confirmed their roles in the HupA biosynthetic pathway. LDC and CAO are found in both eukaryotes and prokaryotes, including plants, mammals, bacteria, yeast, and fungi. In this study, we established a genetic expression system in *C*. *gloeosporioides* capable of high degrees of stable HupA expression. Additionally, we successfully cloned and expressed the *CgLDC* and *CgCAO* genes in *Escherichia coli* and identified the activities of CgLDC and CgCAO associated with HupA biosynthesis. Our results provide valuable insights into genetic modification of strains for selective overexpression of biosynthetic enzymes.

## Results

### Prokaryotic expression of CgLDC and CgCAO and protein purification

**Figure 1:**
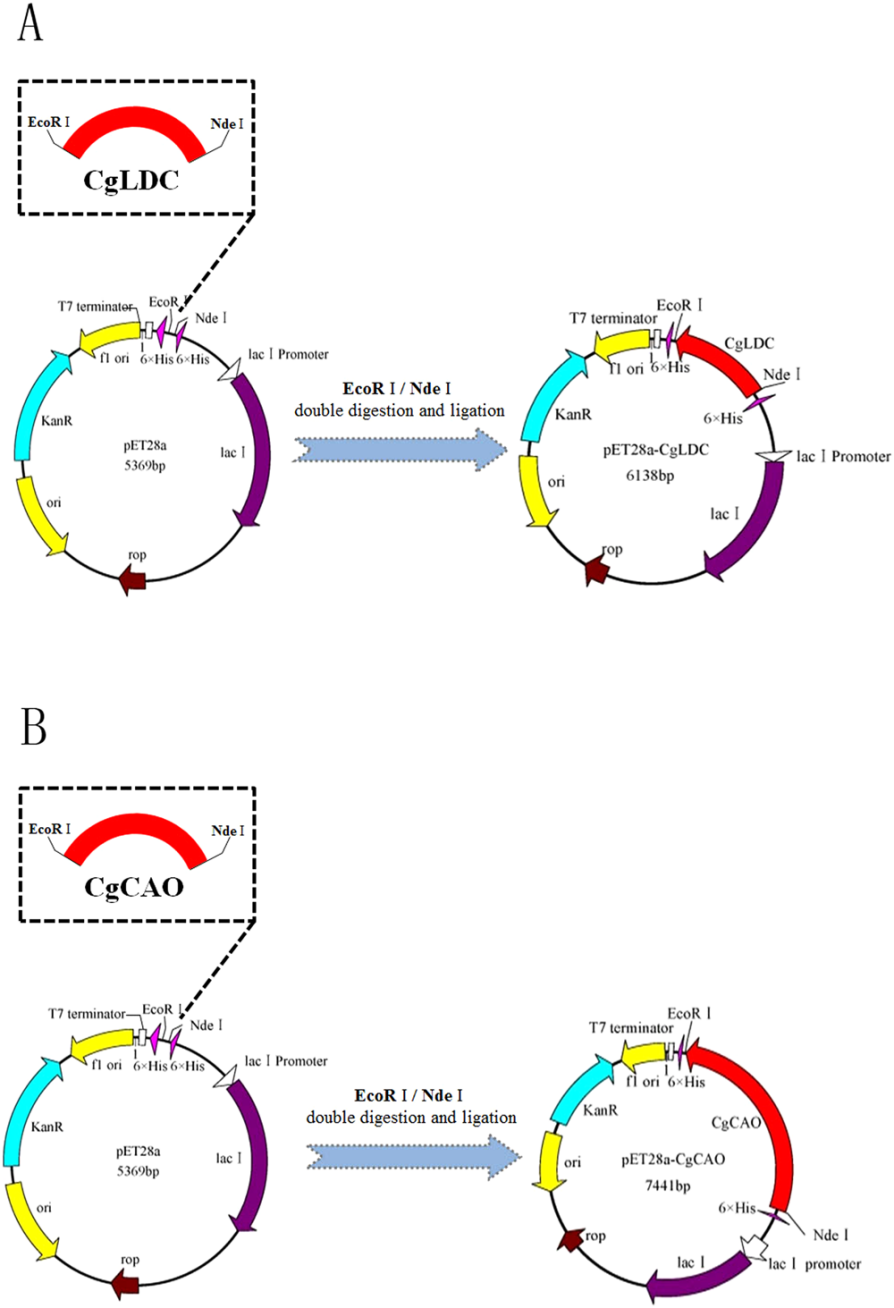
Construction of expression plasmids. (**A**) Construction of the expression plasmid pET28a-CgLDC. (**B**) Construction of the expression plasmid pET28a-CgCAO.

**Figure 2:**
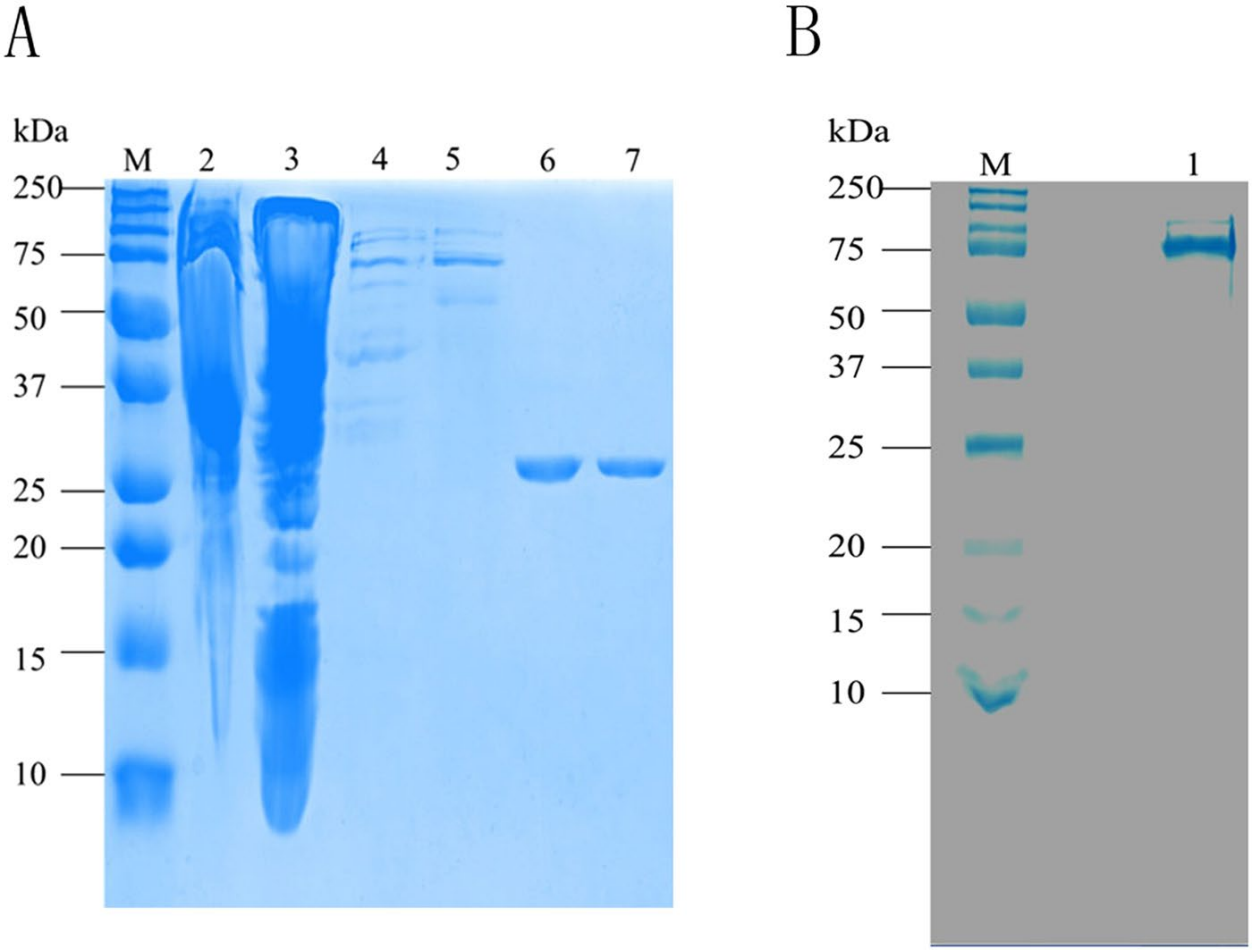
SDS-PAGE analysis of recombinant CgLDC and CgCAO purified by Ni- affinity chromatography. (**A**) SDS-PAGE analysis of recombinant CgLDC. Molecular mass marker (M), supernatant (lane 2), precipitant (lane 3), cell lysate of *Escherichia coli* BL21(DE3)-pET28a-CgLDC (lanes 4 and 5), and purified CgLDC (lanes 6 and 7). (**B**) SDS-PAGE analysis of recombinant CgCAO. Molecular mass marker (M), purified CgCAO (lane 1).

cDNA fragments of *CgLDC* (769 bp) and *CgCAO* (2072 bp) were obtained and cloned from *C*. *gloeosporioides* ES026 into the pET28a vector to yield the expression plasmids pET28a-CgLDC and pET28a-CgCAO (Fig. 1). The recombinant proteins CgLDC and CgCAO with hexahistidine-tags at their respective C-terminal regions were expressed in *E*. *coli* BL21 (DE3) cells and purified to homogeneity using Ni-affinity chromatography. The purified proteins migrated as a single band according to SDS-PAGE analysis in agreement with predicted molecular masses of 28 kDa and 76 kDa, respectively (Fig. 2).

### *In vitro* enzyme assays

**Figure 3:**
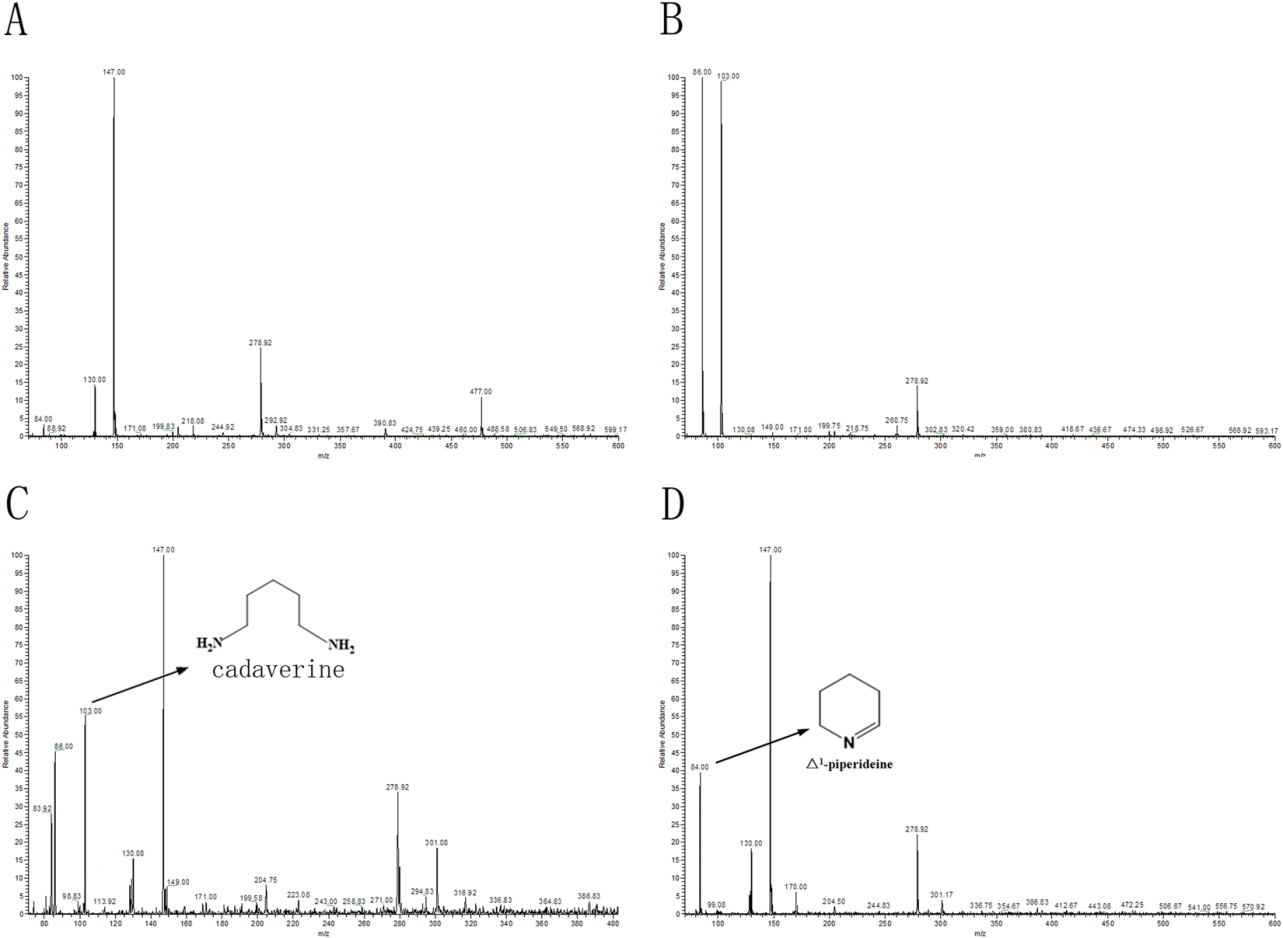
LC-MS analysis of products. (**A**) LC-MS analysis of L-lysine standard. (**B**) LC-MS analysis of cadaverine standard. (**C**) LC-MS analysis of enzymatic formation of cadaverine from L-lysine by CgLDC. Ion chromatograms extracted with *m/z* 103. (**D**) LC-MS analysis of enzymatic formation of Δ^1^-piperideine from cadaverine by CgCAO. Ion chromatograms extracted with *m/z* 84.

**Figure 4:**
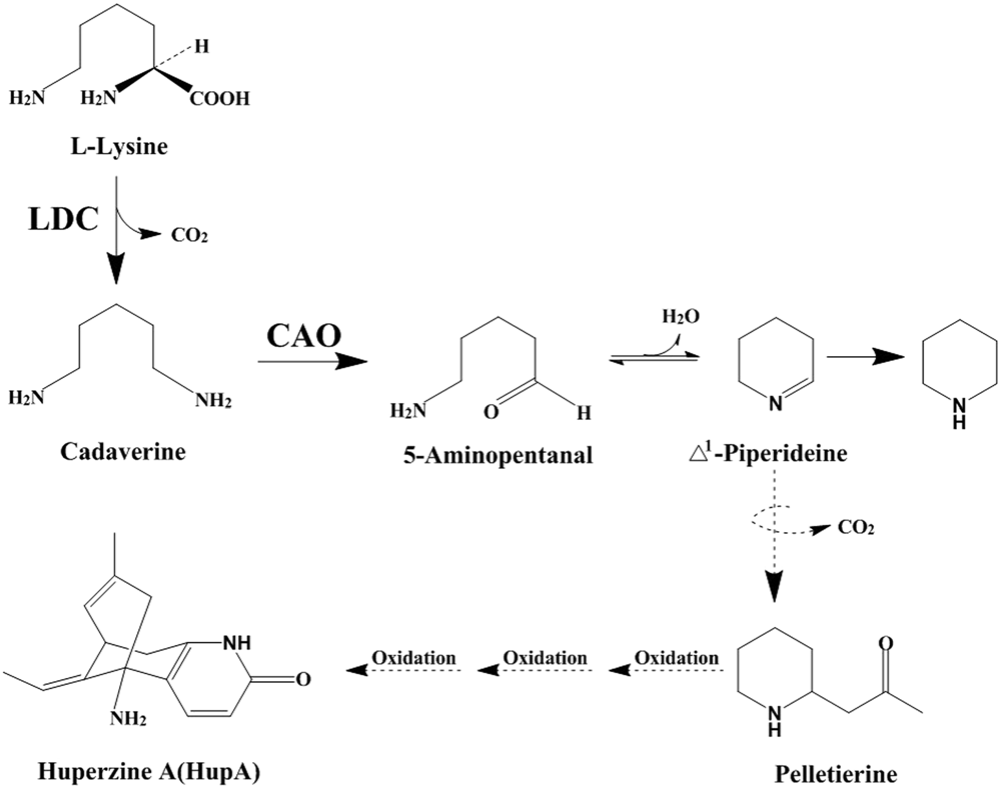
The proposed biosynthetic pathway from L-lysine to Δ^1^-piperideine. LDC: Lysine decarboxylase; CAO: Copper amine oxidase.

LC-MS analysis revealed that CgLDC catalyzed the conversion of L-lysine to cadaverine, and CgCAO converted cadaverine to 5-aminopentanal (Fig. 3), which is the putative biosynthetic precursor of HupA (Fig. 4). By contrast, no catalytic activity was detected from the inactive forms of CgLDC or CgCAO. These results suggested possible CgLDC and CgCAO involvement in HupA biosynthesis.

### Transformation of *C*. *gloeosporioides* ES026 and qRT-PCR analysis

**Figure 5:**
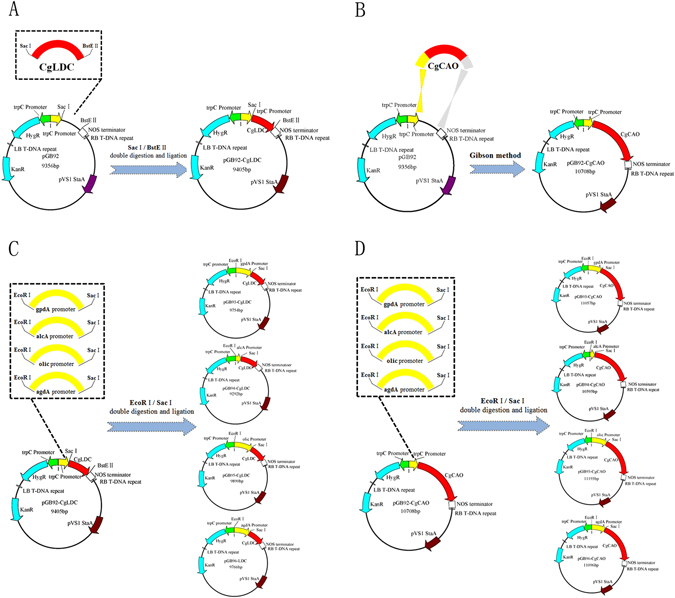
Construction of plasmids overexpressing *CgLDC* and *CgCAO*. (**A**) *CgLDC* was cloned into the pGB92 vector with trpC promoter between restriction sites SacI and BstEI to obtain the recombinant plasmid pGB92-CgLDC. (**B**) The Gibson method was used to construct the recombinant plasmid pGB92-CgCAO. (**C**) The gpdA, alcA, olic, and agdA promoters were ligated into the recombinant plasmid pGB92-CgLDC to produce pGB93-CgLDC, pGB94-CgLDC, pGB95-CgLDC, and pGB96-CgLDC. (**D**) The gpdA, alcA, olic, and agdA promoters were ligated into the recombinant plasmid pGB92-CgCAO to produce pGB93-CgCAO, pGB94-CgCAO, pGB95-CgCAO, and pGB96-CgCAO.

**Figure 6:**
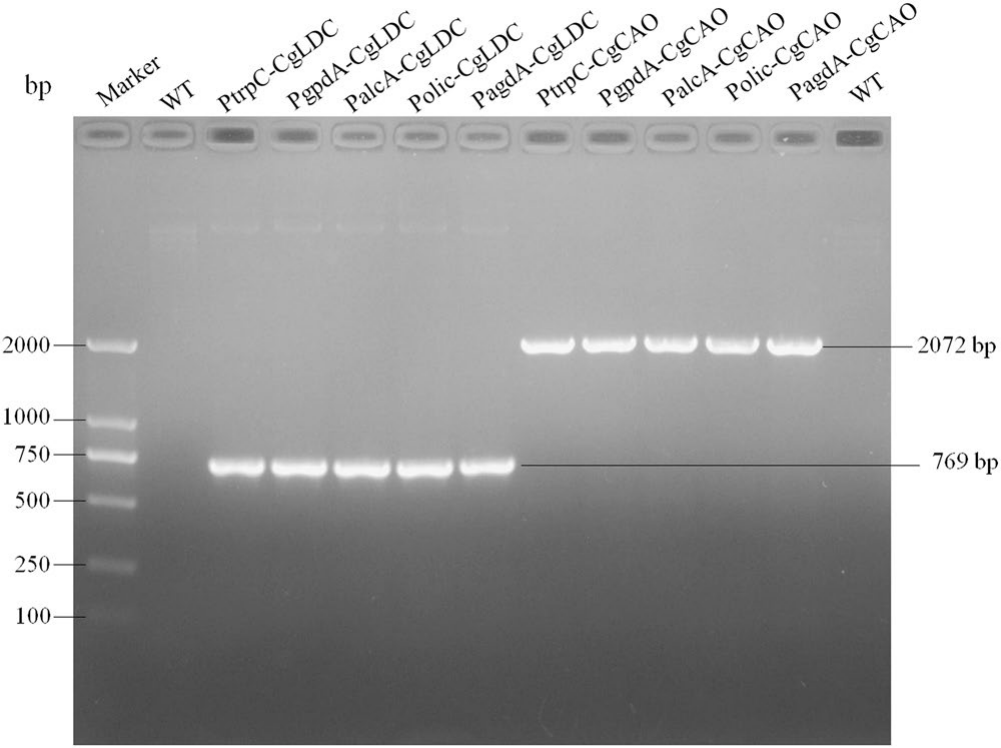
Identification of transformants by PCR.

**Figure 7:**
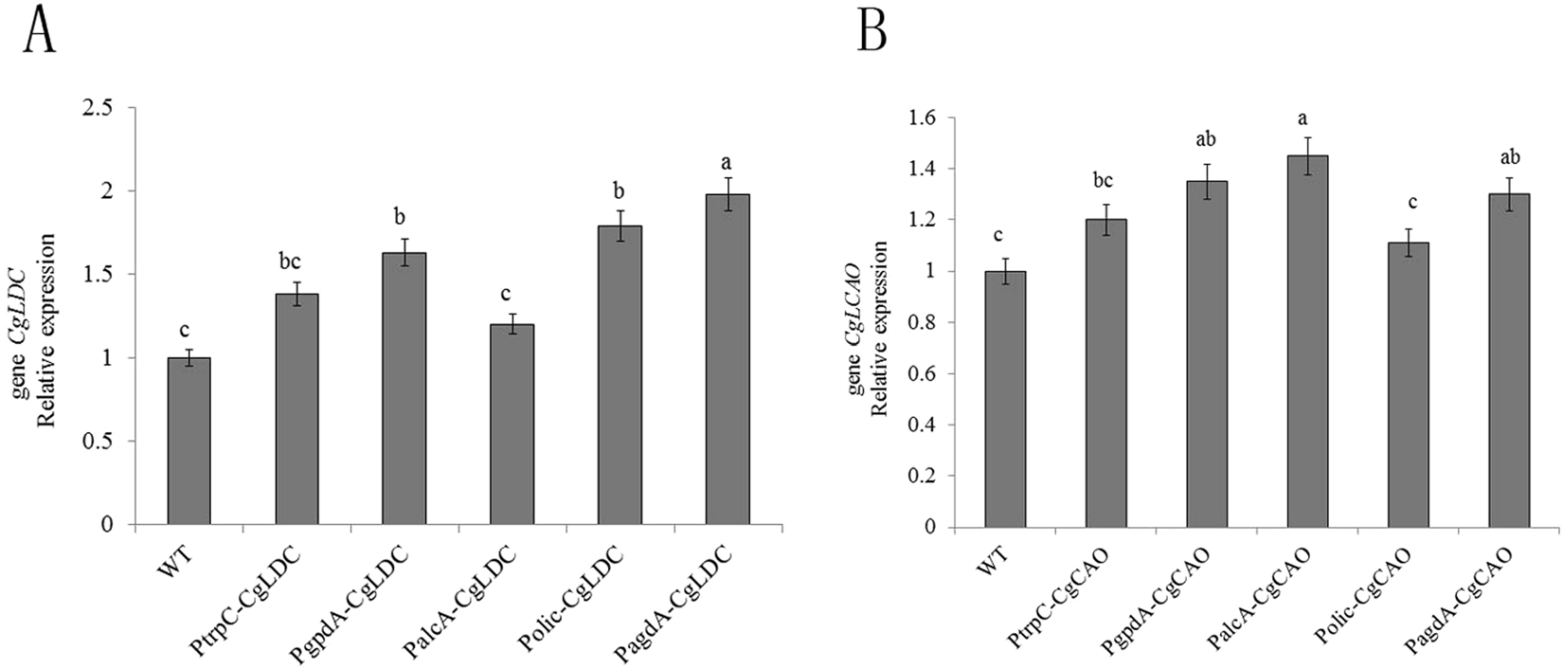
Relative gene expression levels in *C*. *gloeosporioides* ES026 and transformed *C*. *gloeosporioides* ES026 after a 5-day culture. (**A**) Relative expression *CgLDC* in *C*. *gloeosporioides* ES026 and in PtrpC-CgLDC, PgpdA-CgLDC, PalcA-CgLDC, Polic-CgLDC, and PagdA-CgLDC transformants using different promotors after 5 days. (**B**) Relative expression of *CgCAO* in *C*. *gloeosporioides* ES026 and PtrpC-CgCAO, PgpdA-CgCAO, PalcA-CgCAO, Polic-CgCAO, and PagdA-CgCAO transformants using different promotor after 5 days. *Duncan’s multiple range test; *p* < 0.01.

To validate the relationship between *CgLDC* and *CgCAO* expression and HupA production, 10 *CgLDC-* and *CgCAO-*overexpressing plasmids containing different promoters were constructed (Fig. 5). According to methods used for *Agrobacterium* transformation, *C*. *gloeosporioides* ES026 was transformed using the 10 plasmids, and a randomly selected transformant was confirmed by PCR. Our results indicated amplification of appropriately sized DNA fragments (769 bp and 2072 bp, Fig. 6), verifying *C*. *gloeosporioides* ES026 genetic transformation. Quantification by qRT-PCR of *CgLDC* and *CgCAO* expression during fermentation indicated that the PagdA-CgLDA and PalcA-CgCAO transformants exhibited the highest expression levels (Fig. 7).

### Measurement of HupA production

**Figure 8:**
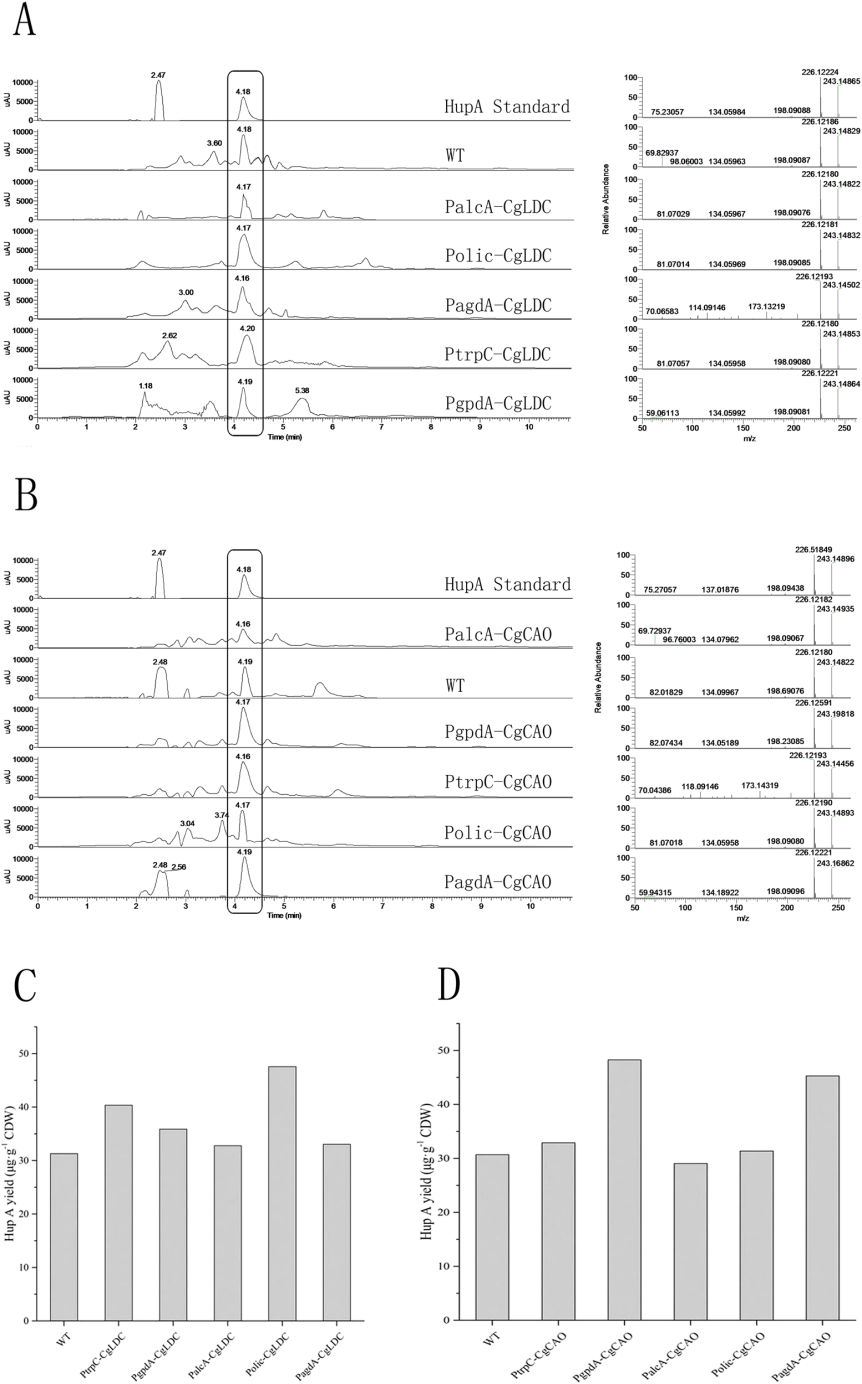
HupA yield from *C*. *gloeosporioides* ES026 and *C*. *gloeosporioides* ES026 transformants. (**A**) LC-HRMS analysis results of wild-type *C*. *gloeosporioides* ES026 and PtrpC-CgLDC, PgpdA-CgLDC, PalcA-CgLDC, Polic-CgLDC, and PagdA-CgLDC transformants. (**B**) LC-HRMS analysis results of HupA yields from wild-type *C*. *gloeosporioides* ES026 and PtrpC-CgCAO, PgpdA-CgCAO, PalcA-CgCAO, Polic-CgCAO, and PagdA-CgCAO transformants. (**C**) HupA yields from wild-type *C*. *gloeosporioides* ES026 and PtrpC-CgLDC, PgpdA-CgLDC, PalcA-CgLDC, Polic-CgLDC, and PagdA-CgLDC transformants. (**D**) HupA yields from wild-type *C*. *gloeosporioides* ES026 and PtrpC-CgCAO, PgpdA-CgCAO, PalcA-CgCAO, Polic-CgCAO, and PagdA-CgCAO transformants.

To investigate transformant effects on HupA production, HupA yield associated with all mutants was analyzed by LC-HRMS. Our results showed that different expression levels of *CgLDC* and *CgCAO* produced different HupA yields; however, high levels of *CgLDC* and *CgCAO* expression resulted in higher yields of HupA, although transformants exhibiting the highest expression levels did not produce the highest yields of HupA. Two genetically altered strains (Polic-CgLDC and PgpdA-CgCAO) yielded stable, high-yielding HupA production (Fig. 8). Our findings revealed that CgLDC and CgCAO were involved in HupA biosynthesis, but that the HupA-synthesis pathway was regulated by separate enzymes.

### Physicochemical properties of CgLDC and CgCAO

*C*. *gloeosporioides* ES026 produced a 28-kDa CgLDC protein containing 256 amino acids, with a predicted formula of C_2662_H_4441_N_885_O_1112_S_230_. The theoretical pI of CgLDC was 5.06, and the instability index (II) was 48.51, indicating a potentially unstable protein. *C*. *gloeosporioides* ES026 produced a 76-kDa CgCAO protein containing 672 amino acids, with a predicted formula of C_3416_H_5274_N_920_O_1019_S_21_. The theoretical pI of CgCAO was 5.60, and the instability index (II) was 39.05, indicating a stable protein.

## Discussion

AD affects millions of people worldwide and is among the four principal death-causing diseases, including heart disease, cancer, and stroke. HupA isolated from *H*. *serrata* is a natural acetylcholinesterase inhibitor used to treat AD. As mentioned in the introduction, very few biosynthetic studies have been performed with HupA, although no investigations have been reported that have attempted to identify the biosynthetic pathway leading directly to HupA, two enzymes (LDC and CAO) have been proposed as the entry point enzymes into the pathway to the HupA^22, 23^. However, work on that enzymes have only been performed in nonrelated taxa^24^. Nevertheless, the feeding that catalyze key transformations in the biosynthesis of HupA and other Lycopodium alkaloids. In this study, next-generation sequencing and *de novo* RNA sequencing of *C*. *gloeosporioides* ES026 was performed, and based on transcriptome analyses by Ma *et al*. and Luo *et al*.^22, 25, 26^, the HupA biosynthetic pathway was investigated. HupA biosynthesis involves primary and secondary enzyme conversion, initiating with acetyl-CoA and biotin and ending with the development of L-lysine, followed by secondary metabolism involving the development of cadaverine. LDC converts L-lysine to cadaverine, and CAO converts cadaverine to 5-aminopentanal and piperideine. Sun *et al*.^27^ cloned *CAO* genes from *H*. *serrata*, and Du Zhu *et al*.^28^ cloned *LDC* genes into the endophytic fungus *Shiraia* sp. Slf14 from *H*. *serrata*, enabling verification of specific characteristics related to LDC and CAO biosynthesis of lycopodium alkaloids. Pelletierine, which is a precursor, is also converted, resulting in HupA synthesis. HupA biosynthesis involves LDC as the first enzyme and CAO as the second enzyme, with LDC transforming L-lysine to cadaverine, and CAO transforming cadaverine to 5-aminopentanal in lycopodium alkaloid biosynthesis. According to Kyoto Encyclopedia of Genes and Genomes analysis, there is only one pathway involved in synthesizing 5-aminopentanal catalyzed by LDC and CAO.

Recombinant plasmids using different promoters to overexpress *CgLDC* and *CgCAO* in *C*. *gloeosporioides* ES026 were constructed, and their expression was determined by qRT-PCR. Additionally, the differential expression of key enzymes involved in HupA biosynthesis and associated metabolic pathways were analyzed, with results indicating that elevated expression of CgLDC and CgCAO produced increased levels of HupA as compared with wild-type *C*. *gloeosporioides* ES026.

In this study, according to the *C*. *gloeosporioides* ES026 genome analysis, CgLDC and CgCAO from *C*. *gloeosporioides* ES026 are unique genes, were investigated for their conversion of L-lysine to 5-aminopentanal in HupA biosynthesis. CgCAO is different from HsCAO^18^, which can produce 5-aminopentanal. Our results indicated that these enzymes could be efficiently expressed in *C*. *gloeosporioides*, that the resulting CgLDC was capable of cadaverine production, and the resulting CgCAO was capable of 5-aminopentanal production, both of which are HupA biosynthetic intermediates, but *In vitro*, this reaction may be weak, experiments are needed to investigate whether CgLDC and CgCAO have similar properties to other LDCs and CAOs. These findings revealed that genetic modification of *C*. *gloeosporioides* ES026 resulted in a variant capable of stable, high-yield production of HupA. Furthermore, we observed that transformants yielding the highest expression of LDC and CAO did not produce the highest yields of HupA, which might have been due to interference by other enzymes involving in HupA biosynthesis. Further investigation is required to elucidate additional details regarding the pathways involved in HupA biosynthesis.

## Materials and Methods

### Fungal strains and plasmids

The strain *C*. *gloeosporioides* ES026, which produced the highest amount of HupA, was isolated from *H*. *serrata* and preserved at the China Center for Type Culture Collection (CCTCC No. 2011046; Wuhan, China). *E*. *coli* BL21 and DH10B cells were cultured in Luria broth (LB) at 37 °C. The plasmid pET28a containing the kanamycin-resistance gene was used as an assisting plasmid for the transformation of *E*. *coli* BL21 cells. Plasmids pGB92, pGB93, pGB94, pGB95, and pGB96 containing the hygromycin B resistance were used as assisting plasmids for the transformation of *C*. *gloeosporioides* ES026.

### *CgLDC* and *CgCAO* expression in *E*. *coli* BL21 (DE3) cells and protein purification

According to the *C*. *gloeosporioides* ES026 genome sequence and transcriptome analysis^21^, the coding regions of *CgLDC* and *CgCAO* were amplified by polymerase chain reaction (PCR) from *C*. *gloeosporioides* ES026 genomic DNA. PCR products were purified using a gel-extraction kit (Omega Bio-tek, Norcross, GA, USA) and cloned into the pET-28a vector between the *EcoR*I and *Nde*I restriction sites to create plasmids pET28a-CgLDC and pET28a-CgCAO for production and purification of the target proteins. The plasmids expressed recombinant proteins containing a hexahistidine-tag at the C-terminus. Subsequently, pET28a-LDC and pET28a-CAO were transformed into *E*. *coli* BL21 cells via heat shock, and transformants were verified by PCR and restriction-enzyme digestion.

Cells were cultured to an OD_600_ of between 0.6 and 0.8 in LB medium containing 100 µg/mL kanamycin at 37 °C and shaking at 200 rpm. Isopropyl β-D-1-thiogalactopyranoside and CuSO_4_ were added to the culture medium to a final concentration of 0.1 mM and 50 µM, respectively, to induce the expression of recombinant CgLDC and CgCAO. The induced broth was maintained at 16 °C with shaking at 200 rpm for an additional 16 h. Cells were collected by centrifugation at 4 °C at 5000 g for 5 min, resuspended in buffer A [50 mM Tris-HCl, 300 mM NaCl, and 4 mM 2-mercaptoethanol (pH 7.6)], and lysed by sonication. Lysates were then centrifuged at 12,000 g for 30 min, and the supernatant was loaded onto a Ni-NTA resin column. Recombinant CgLDC and CgCAO proteins were eluted with buffer B [50 mM Tris–HCl, 300 mM NaCl, 4 mM 2-mercaptoethanol, and 500 mM imidazole (pH 7.6)], and the sizes of the purified proteins were analyzed by sodium dodecyl sulfate polyacrylamide gel electrophoresis (SDS-PAGE)^18^.

### Detection of CgLDC and CgCAO activity

The CgLDC reaction mixture was prepared according to methods reported by Qiao *et al*.^29^. The reaction contained 1.46 mg L-lysine, 1 mg/mL purified recombinant CgLDC, and 40 µg pyridoxal phosphate in 0.1 mM potassium phosphate buffer (pH 8.0). The mixture was incubated at 37 °C for 45 min prior to adding 30 µL HCl to stop the reaction. The same reaction containing boiled (inactive) CgLDC was used as the negative control. Reaction products were extracted with chloroform and analyzed by liquid chromatography mass spectrometry (LC-MS; Column: Thermo Hypersil GOLD aQ column, 150 mm × 2.1 mm, operation of the mass spectrometer was in electrospray positive ion mode. The MS source and chamber conditions were optimised to give maximum analyte signal intensity as follows: Spray voltage: +3500 V; Capillary Temperature: 320 °C; Sheath Gas: 30.0 psi; Aux Gas: 5.0 psi. Probe Heater Temperature: 300 °C; Scan Range: 50–600 m/z; Scan Rate: 1 Hz), gradient conditions with mobile phases of H_2_O and acetonitrile, both containing 1% acetic acid: 0–2 min, 95% H_2_O; 2–9 min, linear gradient from 95% to 60% H_2_O; 9–11 min, 60% H_2_O, 11–14 min, 60% to 95% H_2_O, 14–17 min, 95% H_2_O, and at a flow rate of 0.2 mL/min.

The CgCAO reaction mixture was prepared according to methods reported by Sun *et al*.^30^. The reaction contained 1 mM cadaverine and 0.6 mg/mL purified recombinant CgCAO in 50 mM Tris–HCl buffer (pH 8.0) at a final volume of 500 µL. The mixture was incubated at 25 °C for 16 h, and a separate reaction with boiled (inactive) CgCAO was used as the negative control. Reaction products were extracted with methanol and separated and analyzed by LC-MS.

### *CgLDC* and *CgCAO* overexpression in *C. gloeosporioides* ES026

*CgLDC-* and *CgCAO*-overexpressing plasmids contained different promoters. First, the *CgLDC* gene was cloned into the pGB92 vector along with a trpC promoter located between the *Sac*I and *BstE*I restriction sites to create the recombinant plasmid pGB92-CgLDC, whereas the recombinant plasmid pGB92-CgCAO was constructed according to the Gibson method^31^. Next, The gpdA, alcA, olic, and agdA promoters were amplified by PCR from the pGB93, pGB94, pGB95, and pGB96 vectors, respectively, and inserted into the trpC-CgLDC and trpC-CgCAO plasmids between the *EcoR*I and *Sac*I restriction sites to create the following ten plasmids: pGB92-CgLDC, pGB93-CgLDC, pGB94-CgLDC, pGB95-CgLDC, pGB96-CgLDC, pGB92-CgCAO, pGB93-CgCAO, pGB94-CgCAO, pGB95-CgCAO, and pGB96-CgCAO.

### Transformation of *C*. *gloeosporioides* ES026

*Agrobacterium*-mediated transformations were performed according to the methods of Li *et al*. and Gong *et al*.^31, 32^, with some modifications. Bacterial cultures were diluted to OD_*600*_ = 0.3 using induction medium (IM) containing 200 mM acetosyringone (AS) and were mixed 1:1 with a conidial suspension (10^6^ spores mL^−1^) spread over glass paper on a Co-IM plate containing 400 mM AS. After co-cultivation at 25 °C for 36 h, the membranes were removed, inverted, and placed mycelia-side down onto potato dextrose agar (PDA) plates containing 200 μg/mL cephalosporin to counter-select bacteria and 200 μg/mL hygromycin-B to select for *C*. *gloeosporioides* transformants. After incubation at 25 °C for 3 to 5 days, transformed colonies were transferred to PDA plates for the second round of selection. Each transformant just transferred one recombinant plasmid. The transformants were verified by genomic DNA extraction and PCR identification.

### RNA isolation and quantitative real-time reverse transcription PCR (qRT-PCR) analysis

Total RNA was extracted from 1 g mycelia from *C*. *gloeosporioides* ES026 cultured for 5 days using the RNeasy plant mini kit (Qiagen, Hilden, Germany) according to manufacturer instructions. A PrimeScript reagent kit with gDNA Eraser (TaKaRa Clontech, Shiga, Japan) was used for cDNA synthesis according to manufacturer’s protocol. qRT-PCR was performed in 96-well plates using the StepOne Plus real-time PCR system (Applied Biosystems, Darmstadt, Germany). Each reaction mixture consisted of 12.5 µL 2× SYBR Taq mix (TaKaRa Clontech), 1 µL of forward and reverse primers (10 pmol each), 25 ng of the cDNA, and diethylpyrocarbonate water to a final volume of 25 µL. The oligonucleotide PCR primer pairs are listed in Table 1. The cycling program involved an initial cycle at 94 °C for 30 s, followed by 40 cycles of denaturation at 94 °C for 5 s and annealing and extension at 60 °C for 50 s. mRNA expression levels of the target genes were normalized to the mRNA expression level of the reference gene *Tubalin*. Comparative expression levels were measured by the 2^−ΔΔCT^ technique using StepOne version 2.3 and DataAssist software (Applied Biosystems).

**Table 1:** Primers used for qRT-PCR.

Gene name	Primer name	Sequence(5′-3′)
*CgLDC*	CgLDC-F	TCATATGGTAGCCGCCGAC
	CgLDC-R	CACCTCCGTAGACCAGATCA
*CgCAO*	CgCAO-F	GATCTACGGAACTGACGGTAT
	CgCAO-R	CGCTGGTTGAAGCCGATGAAG
*Tubalin*	Tubalin-F	CTTGCTCTTCTTGCCATAGTCG
	Tubalin-R	CCTTCAGGGCTTCCTCGTCT

### Measurement of HupA production

Transfer of transformant solution was performed according to the method of Zhao *et al*.^33, 34^ with minor modifications. Fermented mycelia were collected by centrifugation at 12,000 g or 10 min, followed by drying at 40 °C overnight and grounding into powder. For chemical extraction, each sample of raw material (1 g) was produced using 0.5% HCl [(30 mL (w/v)] overnight, followed by ultrasonication in a water bath at 40 °C for 1 h. The ingredients were then filtered, and the filtrates were rendered with ammonia solution to pH 9.0. After 1 h, the water phase was extracted three times with chloroform, and the combined chloroform extracts were evaporated to dryness *in vacuo*. The dry residue was mixed with 1 mL methanol, passed through a 0.45-µm polytetrafluoroethylene syringe filter, and analyzed by LC-HRMS (Agilent Zorbax SB-C18; 150 mm × 4.6 mm, 5-µm diameter, operation of the mass spectrometer (MS) was in electrospray positive ion mode. The MS source and chamber conditions were optimised to give maximum analyte signal intensity as follows: Spray voltage: +3500 V; Capillary Temperature: 320 °C; Sheath Gas: 30.0 psi; Aux Gas: 5.0 psi. Probe Heater Temperature: 300 °C; Scan Range: 50–600 m/z; Scan Rate: 1 Hz). The mobile phases consisted of H_2_O and 5% acetonitrile or 100% acetonitrile (65%: 35%) at a flow rate of 0.6 mL/min. Quantification was performed using the standard curve generated from the HupA standard over a concentration range of between 0.5 and 8.0 mg/L, where the peak area and height showed linear correlations with the absorbance (R^2^ = 0.9991).

### Bioinformatics analysis of CgLDC and CgCAO

Physicochemical properties were predicted using the ExPASy-ProtParam tool (http://web.expasy.org/protparam/), and hydrophobic/hydrophilic analysis was performed by ExPASy-ProtScale (http://web.expasy.org/protscale/). Protein signal peptides were predicted using the SignalP 4.1 server (http://www.cbs.dtu.dk/services/SignalP/), and transmembrane regions were predicted using the TMHMM server version 2.0 (http://www.cbs.dtu.dk/services/TMHMM/). Protein subcellular localization was predicted by ProtComp version 9.0 (http://linux1.softberry.com/berry.phtml?topic=protcompan&group=programs&subgroup=proloc).
